# HMGB1-triggered inflammation inhibition of notoginseng leaf triterpenes against cerebral ischemia and reperfusion injury via MAPK and NF-κB signaling pathways

**DOI:** 10.3390/biom9100512

**Published:** 2019-09-20

**Authors:** Weijie Xie, Ting Zhu, Xi Dong, Fengwei Nan, Xiangbao Meng, Ping Zhou, Guibo Sun, Xiaobo Sun

**Affiliations:** 1 Beijing Key Laboratory of Innovative Drug Discovery of Traditional Chinese Medicine (Natural Medicine) and Translational Medicine, Institute of Medicinal Plant Development, Peking Union Medical College and Chinese Academy of Medical Sciences, Beijing 100193, China; xwjginseng@126.com (W.X.); ginseng123@163.com (T.Z.); dx5212004@126.com (X.D.); pumchNFW@163.com (F.N.); xbmeng@implad.ac.cn (X.M.); zhoup0520@163.com (P.Z.); 2 Key Laboratory of Bioactive Substances and Resources Utilization of Chinese Herbal Medicine, Ministry of Education, Institute of Medicinal Plant Development, Chinese Academy of Medical Sciences & Peking Union Medical College, Beijing 100193, China; 3 Key Laboratory of Efficacy Evaluation of Chinese Medicine against Glycolipid Metabolic Disorders, State Administration of Traditional Chinese Medicine, Institute of Medicinal Plant Development, Peking Union Medical College and Chinese Academy of Medical Sciences, Beijing 100193, China; 4 Zhongguancun Open Laboratory of the Research and Development of Natural Medicine and Health Products, Institute of Medicinal Plant Development, Chinese Academy of Medical Sciences & Peking Union Medical College, Beijing 100193, China; 5 Key Laboratory of new drug discovery based on Classic Chinese medicine prescription, Chinese Academy of Medical Sciences, Beijing 100193, China

**Keywords:** notoginseng leaf triterpenes, HMGB1, cerebral ischemia and reperfusion injury, inflammation, MAPK, NF-κB

## Abstract

Ischemic stroke is a clinically common cerebrovascular disease whose main risks include necrosis, apoptosis and cerebral infarction, all caused by cerebral ischemia and reperfusion (I/R) injury. This process has particular significance for the treatment of stroke patients. Notoginseng leaf triterpenes (PNGL), as a valuable medicine, have been discovered to have neuroprotective effects. However, it was not confirmed that whether PNGL may possess neuroprotective effects against cerebral I/R injury. To explore the neuroprotective effects of PNGL and their underlying mechanisms, a middle cerebral artery occlusion/reperfusion (MCAO/R) model was established. In vivo results suggested that in MCAO/R model rats, PNGL pretreatment (73.0, 146, 292 mg/kg) remarkably decreased infarct volume, reduced brain water content, and improved neurological functions; moreover, PNGL (73.0, 146, 292 mg/kg) significantly alleviated blood-brain barrier (BBB) disruption and inhibited neuronal apoptosis and neuronal loss caused by cerebral I/R injury, while PNGL with a different concertation (146, 292 mg/kg) significantly reduced the concentrations of IL-6, TNF-α, IL-1 β, and HMGB1 in serums in a dose-dependent way, which indicated that inflammation inhibition could be involved in the neuroprotective effects of PNGL. The immunofluorescence and western blot analysis showed PNGL decreased HMGB1 expression, suppressed the HMGB1-triggered inflammation, and inhibited microglia activation (IBA1) in hippocampus and cortex, thus dose-dependently downregulating inflammatory cytokines including VCAM-1, MMP-9, MMP-2, and ICAM-1 concentrations in ischemic brains. Interestingly, PNGL administration (146 mg/kg) significantly downregulated the levels of p-P44/42, p-JNK1/2 and p-P38 MAPK, and also inhibited expressions of the total NF-κB and phosphorylated NF-κB in ischemic brains, which was the downstream pathway triggered by HMGB1. All of these results indicated that the protective effects of PNGL against cerebral I/R injury could be associated with inhibiting HMGB1-triggered inflammation, suppressing the activation of MAPKs and NF-κB, and thus improved cerebral I/R-induced neuropathological changes. This study may offer insight into discovering new active compounds for the treatment of ischemic stroke.

## 1. Introduction

Ischemic stroke, also known as cerebral infarction, is one of the leading causes of death with substantial morbidity and mortality worldwide. Nearly 6.2 million people die from stroke each year, and it is estimated that the lifetime risk for stroke is 8% to 10%. Ischemic stroke accounts for 85% of all strokes [1]. Ischemia causes brain infarction, however. Moreover, the subsequent reperfusion phase results in brain injury, including BBB disruption, hemorrhagic transformation, and massive brain edema, which is involved in a wide range of neuropathic alteration and processes including oxidative stress, inflammatory stress [2] and cytokine damage glutamate toxicity, Ca^2+^ overload, excessive nitric oxide synthesis, and apoptosis [1,3,4]. Although the mechanisms of cerebral ischemia and reperfusion I/R injury are complex and involve the interaction of numerous pathophysiological processes, there are accumulating evidences that inflammation and apoptosis are involved [5,6,7,8].

The high mobility group box-1 protein (HMGB1), as a nuclear DNA-binding protein and an important damage associated molecular pattern (DAMP), is released from necrotic and dying neural cells in the ischemic brain, leading to the activation of microglia and the expression of inflammatory factors in ischemic brain, and it may activate TLR 2/4 and RAGEs signaling following rapid translocation to the cytoplasm or release from dying cells after cerebral ischemia [9,10], which can promote the activation of inflammatory responses. Sequentially, the signalosome comprising inflammatory response, triggered by HMGB-1/TLR4, can stimulate nuclear factor kappa B (NF-κB) translocation. Moreover, the TRAF6–IRAK1–TAK1 complex triggers mitogen-activated protein kinase (MAPK) phosphorylation including p38, JNK, and ERK, which play key roles in inflammation [11,12,13,14,15]. The activated MAPKs mainly function as mediators of cellular stress by phosphorylating intracellular enzymes, transcription factors, and cytosolic proteins involved in cell survival, inflammatory mediators production, and apoptosis [16]. Accordingly, the therapeutic strategy associated with HMGB-1/TLR4 signaling might represent a promising approach to the restriction of neuro-inflammatory processes and the amelioration of cerebral stroke damage [10,17,18,19]. Development of an effective anti-inflammatory drug may be an efficient approach for the treatment of ischemic stroke-induced brain injury [10,16,20]. However, most of these treatments have disappointingly been found to be ineffective during the acute phase of stroke [21]. Furthermore, many anti-neuro-inflammatory drugs show poor outcomes for the treatment of ischemic stroke in clinical trials. Hence, development of new and effective neuroprotective agents for ischemic stroke is clinically significant and urgently needed.

Traditional Chinese medicines, used in China for thousands of years with high efficiency and low toxicity, have attracted great interest in recent years [22,23,24,25]. *Panax notoginseng (Burk) F. H. Chen* is a commonly used Chinese medicinal herb and plant, the roots and stems of which have been used for the treatment of cardiovascular disease in many Asian countries [22]. Current experimental evidences indicate that *panax notoginseng*, and their extracts, panax notoginseng saponins (PNS) possess many beneficial effects, such as neuroprotective, antiinflammatory, antiapoptotic [26], and anticonvulsant effects in various models [3,24,27,28,29]. Additionally, recent studies have demonstrated that PNS exerts protective effects against cerebral I/R injuries, which are involved in the aspects: anti-oxidant and associated apoptotic effects; anti-inflammatory or immunostimulatory-related effects on apoptosis or necrosis; neurological cell cycle, proliferation, differentiation, and regeneration; and energy metabolism and regulation of cellular ATP levels, BBB permeability, excitatory amino acids, and other processes, including the activation of nerve growth factor, excitotoxicity, and excessive Ca^2+^ influx into neurons [3,24,27,28,29,30,31].

It was known that PNS were mainly obtained and purified from the roots of *panax notoginseng (Burk) F. H. Chen.* However, the *panax notoginseng (Burk) F. H. Chen* stems and leaves were often ignored. Annual production of Panax notoginseng stems and leaves is over 25 million kilograms in China, but its effective utilizing rate is under 5%, so many panax notoginseng resources are wasted. In contrast, panax notoginseng stems and leaves contain high protein, crude fiber, vitamin C and carotenoids, low fat and rich mineral element, among which contents of zinc, iron and manganese are remarkably high. The contents of protein, carotenoids and vitamin C are higher than those of ordinary vegetables, thus, *panax notoginseng* stems and leaves have a higher nutritional value. Its major active ingredients in panax notoginseng stems and leaves are saponins, the chemical structure is mainly protopanaxadiol type that has functions of sedative-hypnotic action, analgesia, blood lipid regulating, anti-inflammatory/retarding the aging process. And panax notoginseng stems and leaves has been confirmed as the raw materials of production of advanced cosmetics, functional foods and common foods [31,32,33,34,35], which deserves further research and development into new products. Currently, notoginseng leaf friterpenes (PNGL), as the total saponins of Panax notoginseng stem and leaf, have already been shown to exert potent neuroprotective and anti-apoptotic properties. However, the beneficial effects of PNGL were rarely detected in nervous diseases, and several reports have revealed its diverse pharmacological properties and raised some speculative proposals concerning its effect mechanisms [31,32,33]. At present, there was no clear evidence on whether PNGL has neuroprotective effects against cerebral I/R injury.

**Figure 1:**
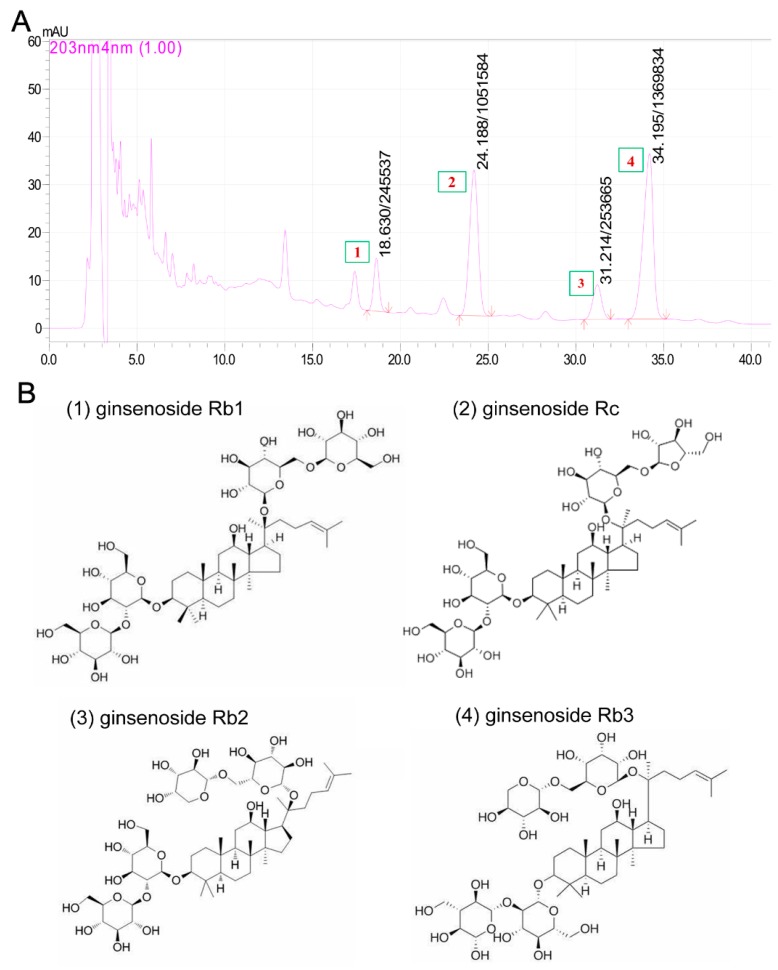
The chromatograms and chemical components of notoginseng leaf triterpenes (PNGL) used in experiments via the high performance liquid chromatography. (**A**) The chromatograms of PNGL samples. (**B**) Chemical component structures, mainly including ginsenoside Rb1 (No.1), ginsenoside Rb2 (No.3), ginsenoside Rb3 (No.4), and ginsenoside Rc (No.2).

Base on the reported neuroprotective effects of PNS against cerebral I/R injuries, we hypothesized that PNGL may have neuroprotective effects of relieving cerebral I/R injury with inhibiting HMGB1-triggered inflammation and apoptosis by regulating the MAPK and NF-κB signaling pathways. Therefore, middle cerebral artery occlusion/reperfusion (MCAO/R)-operated rats were used to explore the effects of the PNGL against cerebral ischemia stroke. In additions, our chemical researchers have found that the content of total saponins of panax notoginseng stems and leaves is 4% to 6%. Among them, monomeric saponins contained are mainly 20(s)-protopanaxadiol saponins, and contains almost no ginseng triol saponins which is the biggest difference between PNS and PNGL. As shown in Figure 1, it mainly contains ginsenoside Rb1, ginsenoside Rb2, ginsenoside Rb3, and ginsenoside Rc. Additionally, eleven batches of PNGL samples were monitored by the chemical fingerprinting assay (Supplementary Material S1).

## 2. Methods and Materials

### 2.1. Animals

Male Sprague Dawley (SD) rats (specific pathogen free, weighing 280–300 g) were used in this study, and they were purchased from Beijing Vital Lihua Experimental Animals Co., Ltd. Rats were housed at an ambient temperature of 20 ± 1 °C and a relative humidity of 55 ± 15% with artificial light for 12 h each day and free access to a standard laboratory chow diet and sterilized drinking water throughout the experiments. All efforts were made to minimize the number of animals used and their suffering. The study was conducted in accordance with the Declaration of Helsinki. The protocol was approved by the Laboratory Animal Ethics Committee of the Institute of Medicinal Plant Development, Peking Union Medical College, and conformed to the Guide for the Care and Use of Laboratory Animals (Permit Number: SYXK 2017-0020).

### 2.2. Experimental Groups and Drug Administration

Rats were divided into seven groups (10 for each group), namely, a sham-operated group, a MCAO/R model group, a 73 mg/kg dose of PNGL group, a 146 mg/kg dose of PNGL group, a 192 mg/kg dose of PNGL group, and a positive drug butylphthalide (NBP, 60 mg/kg) group and an aspirin group (ASP, 10 mg/kg), according to a random number table. The PNGL, NBP, and ASP samples were all dissolved in normal saline. Then, rats were intragastrically administered daily in a constant volume. Moreover the animals in the sham and MCAO/R groups were given an equal volume of saline water. Drug administration groups were exposed to continuous gastric administration (once per day) for 7 days before MCAO/R operations.

### 2.3. Transient Focal Brain Ischemia/Reperfusion Mode

As previously described [3,36], MCAO/R model was employed to induce transient focal brain ischemia/reperfusion. In brief, rats were anaesthetized with Zoletil 50 (Virbac S.A, Carros, France) via intraperitoneal injections. A silicone-coated 3/0 monofilament was introduced into the right internal carotid artery and advanced to occlude the middle cerebral artery for 2 h. Rats in sham-operated group underwent the same procedures except occluding the MCA. The temperature was maintained at 32 ± 0.1 °C until animals woke up completely.

**Figure 2:**
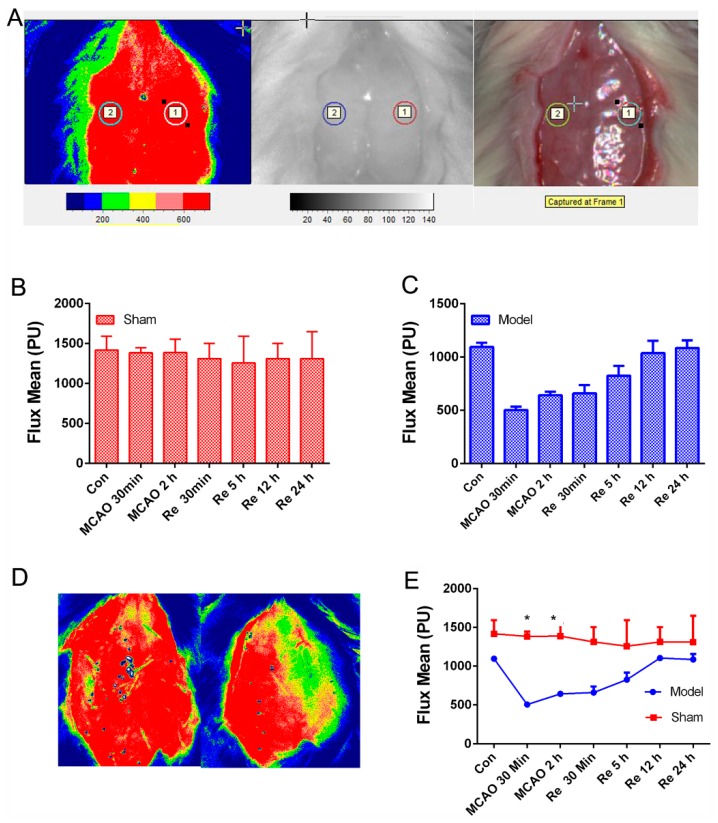
Cerebral blood flow assessed by the laser Doppler blood flow assessment in rats with a middle cerebral artery occlusion/reperfusion MCAO/R injury. (**A**) The monitored place (core cortex, 2 mm posterior and 6 mm lateral to the bregma). (**B**,**C**) Flux mean value of cerebral blood flows in sham and MCAO/R group. (**D**) The images of cerebral blood flow in the sham group (left) and the model group (right). (**E**) Flux mean value at different time points. Mean values ± SEM; * *p* < 0.05, sham group versus MCAO/R group.

As shown in Figure 2A, cerebral blood flow velocity of the right MCA territory (core cortex, 2 mm posterior and 6 mm lateral to the bregma) was assessed by the laser Doppler blood flow assessment (FLPI-2, Moor Instruments, Wilmington, DE). After 2 h, suture was removed to induce blood reperfusion whose flow velocity was monitored again (Figure 2B–E) at different time points (2,5,12,24 h), which contributed to improving the reliability and repeatability of the MCAO/R model.

### 2.4. Measurement of Neurological Deficit

Rats were monitored by video camera after surgery, and the time of death was recorded. Neurological deficits at 22 h after MCAO were assessed using Zea Longa Scores [37,38,39]. Based on Longa Scores, neurological function was graded on a series of scales from 0 to 4 with higher scores indicating more severe neurologic deficits. Tests were independently performed by two investigators blinded to animal grouping.

### 2.5. TTC Staining

As previously described [9,10,36], 2,3,5-triphenyltetrazolium chloride (TTC, Sigma, St. Louis, MO, USA) staining was used to assess the ischemic infarction. After MCAO/R operation, rats were anaesthetized with Zoletil 50 (20 mg·kg^-1^) via intramuscular injection (Virbac S.A, Carros, France). All brains were sliced into 2 mm sections, incubated for 20 min in a 2% solution of TTC (37 °C) temperature, and then fixed in 4% paraformaldehyde. The infarct areas on each slice were quantified by using the ImageJ 1.44p software (National Institutes of Health, Bethesda, MD, USA).

### 2.6. Detection of Brain Water Content

As previously described [9,10,36], a 3-mm section of the ischemic hemisphere brain was cut from the anterior pole to detect the water content in the brain tissue. The wet-dry method was applied to determine brain water content in another subgroup. An electronic scale was used to weigh the ischemic and non-ischemic hemispheres (wet weight). After the ischemic brain hemisphere was dried overnight at 105 °C in a desiccating oven, it was weighed again (dry weight), and the total brain water content was calculated according to previous reports [36].

### 2.7. Measurement of BBB Permeability

BBB integrity and permeability was quantitatively assessed by measuring the extravasation of Evans blue dye, which serves as a marker of albumin leakage. As previously described [36]. Evans blue (2% in saline, 4 mL/kg; Sigma-Aldrich) was administered 90 min before sacrifice followed by transcardially perfused with saline to remove the residual dye from the vessels. The hemispheres were weighed and incubated in dimethyl formamide (DMF, Sigma-Aldrich, Shanghai -Greater China Regional Headquarters, China) in 60 °C water bath overnight. After that, Evans blue content was determined in supernatants at 632 nm and expressed as microgram per gram brain. Gradient concentrations of Evans blue were used to build standard curve.

### 2.8. Detection of Inflammatory Cytokines in Serums and Brain Tissues from Rats

The blood samples from each groups were collected [36,40,41], and then centrifuged at 3000 rpm·min^−1^ for 20 min, the blood serums were obtained and stored at −80 °C for further measurement. Furthermore, the hippocampus and cortex tissues were rapidly removed and carefully dissected on an ice plate, frozen in liquid nitrogen and then stored at −80 °C until the assays were performed. Then, the hippocampus and cortex samples were partly weighed and homogenized by sonication in a specified amount of normal saline (100 microliters/ 10 milligrams). The homogenate was kept at 4 °C for 30 min and then centrifuged at 12,000 rpm for 3 min at 4 °C. The supernatant was collected, obtained and stored at −20 °C and then used to measure the inflammatory cytokines by using assay kits.

The protein concentration of the collected supernatants was determined via the BCA protein assay kit (CWBIO, Beijing, China). The inflammatory cytokines from the ischemic brains and the blood serums were analyzed using ELISA kits (Beijing HaiTai Tong, Da Sci Tech Ltd., Beijing, China). All experimental steps were performed according to the kit operation specifications. OD values were measured by a microplate reader.

### 2.9. Histopathological Examination

Giemsa staining was commonly used to revealed morphological features of injured neurons in the cerebral cortex. As previously described [9,10,36], the samples were embedded in paraffin and cut in 5-μm slices; 5-μm-thick serial coronal sections were generated and mounted on slides. The sections were stained according to the described standard protocol. Images of stained slides were acquired using a light microscope (Leica, Leica DM4000B, Wetzlar, Germany). After 24 h following MCAO/R operation, the brains were processed as above. Samples were embedded in paraffin and cut in 5-μm slices; 5-μm-thick serial coronal sections were generated and mounted on slides. After washed in cold water, the paraffin sections were stained with Giemsa staining for 20 min, rinsed with PBS, dehydrated with graded alcohol, made transparent with xylene, and fixed with neutral glue. An optical microscope was used to observe each section, and images were randomly selected for image analysis via ImageJ software (Media Cybernetics, USA).

### 2.10. TUNEL Staining Assay

To measure the extent of neuronal apoptosis in cerebral I/R injury, an in situ apoptosis detection kit (POD, Roche, and Mannheim, Germany) was employed to discover neuronal apoptosis caused by ischemia. A TUNEL analysis was carried out with minor modification according to the manufacturer’s instructions. Firstly, the sections were installed on the slides and permeabilized by incubating them with 100 µL of 20 µg/mL proteinase K solution for 15 min. Next, the sections were incubated with 100 µL of 0.3% H_2_O_2_ for 5 min and incubated by equilibration buffer and terminal deoxynucleotidyl transferase to inactivate endogenous peroxidases. Then, anti-digoxigenin-peroxidase conjugates were employed to incubate the sections. Finally, the utilization of diaminobenzidine demonstrated peroxidase activity in all tissue sections, and the slices were counterstained with hematoxylin. TUNEL-positive cells were visualized by using a Leica microscope (Leica DM4000B, Germany), and images were randomly selected for image analysis via ImageJ software.

### 2.11. Immunofluorescence

To explore the effects of PNGL on HMGB expression and IBA1-marked microglia activation in CUMS-induced rats [42,43], immunofluorescence staining was performed as previously described [7,8,44,45]. Briefly, after the micro slides were deparaffinized, dewatered, and restored with a citrate-EDTA antigen retrieval solution (P0086, Beyotime, Shanghai, China) for 20 min at 95 ℃, they were cooled down and washed with PBS three times (10 min per time), blocked with 5% goat serum albumin at room temperature for 60 min, and then incubated overnight with an anti-HMGB1 antibody (ab79823, 1:500 in dilution) and IBA1 (ab15690, 1:400 in dilution) at 4 °C. Subsequently, they were incubated with a TRITC-conjugated goat anti-rabbit IgG at a 1:100 dilution (CW0160, CWBIO, Beijing, China) and a Alexa Fluor 488-labeled goat anti-mouse IgG (P0188, Beyotime, Shanghai, China) for 2 h at room temperature, and then counterstained by DAPI (5 μg/mL) for 10 min. Images were observed using fluorescence microscopy (Leica, Germany Q9). The fluorescence intensity was evaluated by the ImageJ 1.44p software.

### 2.12. Western Blot Analysis

Western blotting was performed as previously reported [28,30,36,46]. Based on the manufacturer’s instructions, the hippocampal and cortex tissues were weighed and homogenized using a tissue protein extraction kit (CWBIO, Shanghai, China) supplemented with 1% proteases and a phosphatase inhibitor cocktail (CWBIO, Shanghai, China), and the non-ischemia correspondent hippocampal and cortex tissues were treated as the PNGL control samples. After the homogenates were centrifuged at 12,000×g for 15 min at 4 °C, the supernatant samples including total protein proteins were collected. Then, the protein concentration in the supernatant was determined by a BCA assay (CWBIO, Shanghai, China). Finally, the protein samples (3.5 mg/mL) were diluted with 5 × SDS loading buffer (CWBIO, Shanghai, China), denatured in boiling water for 5 min, and then stored at −80 °C until use. Protein samples were loaded onto the SDS-PAGE gel (10–15%), separated electrophoretically, and transferred onto NC membranes (Millipore, Bedford, MA, USA). After blocking the nonspecific binding sites for 2 h in 5% non-fat milk and Tris-buffered saline (TBS)/Tween 20 at room temperature, the membranes were individually incubated overnight at 4 °C with the following primary antibodies: P44/42 (CST4695, 1:1000), SARK/JNK (CST9525, 1:1000), P38 (CST8690, 1:1000), p-P44/42 (CST4370, 1:1000), p-SARK/JNK (CST4668, 1:1000), p-P38 (CST4511, 1:1000), NF-κB (CST8242, 1:1000), p-NF-κB (CST3033, 1:1000), Cox2 (CST12282, 1:1000), c-Fos (ab134122, 1:5000), Caspase 9 (ab1884768, 1:1000), Cleaved Caspase 3 (ab49822, 1:500), Caspase 8 (ab25901 1:1000), IL-1β (EXP. 1:500), and β-actin (EXP0036

F, 1:2000). Then, the membrane was incubated at room temperature for 2 h with horseradish peroxidase conjugated antibodies at a 1:2000 dilution. Protein expression was detected by an enhanced chemiluminescence method and imaged by using the ChemiDoc XRS instrument (Bio-Rad, Hercules, CA, USA). To eliminate variations in protein expression, three independent experiments were performed.

### 2.13. Data and Statistical Analyses

Data are presented as the mean values ± standard deviation (SD) or standard error of the mean (SEM). All analyses were performed by using GraphPad Prism 8.0 statistical software (GraphPad Software, Inc., La Jolla, San Diego, CA, USA). Two-way analysis of variance (ANOVA) was used with drug (PNGL versus Vehicle) and treatment (MCAO/R vsrsus Control) as independent factors. Group differences after significant ANOVAs were measured by post hoc Bonferroni test, and *p* < 0.05 was considered statistically significant.

## 3. Results

### 3.1. PNGL Improves Neurological Functions and Attenuate Brain Swellings and Infarcts in MCAO/R Rats

**Figure 3:**
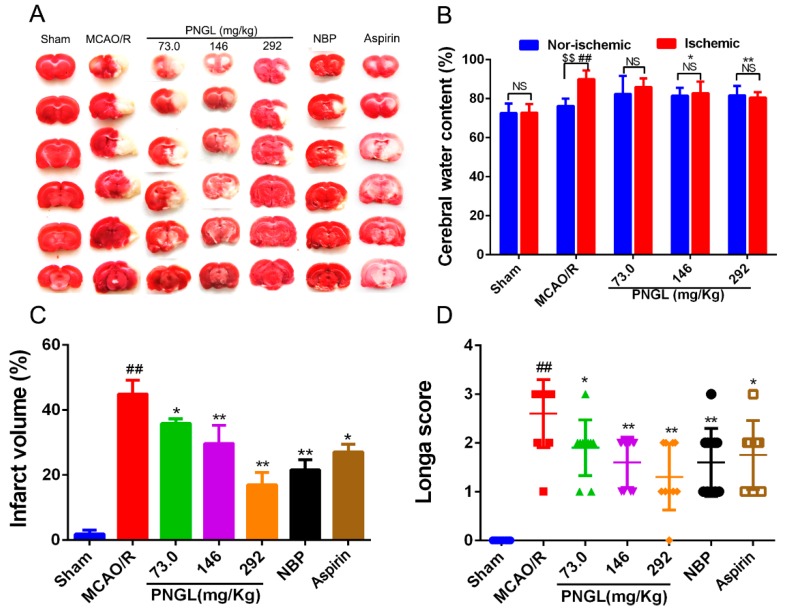
Effects of PNGL on infarct volume, brain water content, and neurological deficit scores in MCAO/R injury rats. PNGL improves neurological functions and attenuate brain swellings and infarcts in MCAO/R rats. (**A**) Representative images of 2, 3, 5-triphenyltetrazolium chloride -stained brain sections from the sham-operated or PNGL-treated animals collected 24 h after infarction; red tissue is healthy; white tissue is infarcted (*n* = 3–6 in each group). (**B**) Brain water content in ischemia hemispheres of all groups (*n* = 4 in each group). (**C**) Quantitative analysis of the infarct volume (n = 3 in each group). (**D**) Neurological deficit scores in all groups (*n* = 10 in each group). Mean values ± SD; * *p* < 0.05, ** *p* < 0.01 versus MCAO/R group; # *p* < 0.05, ## *p* < 0.01, versus sham group; $$*p* < 0.01, Nor-ischemic versus Ischemic; NS means no significance, Nor-ischemic versus Ischemic.

To assess the neuroprotective effects of PNGL on focal brain ischemic injury, SD rats were subjected to 2 h of MCAO and 24 h of reperfusion. Extensive infarction was detected by TTC staining in the cerebral cortical and subcortical areas over a series of sections of the ipsilateral hemisphere in rats subjected to MCAO (Figure 3A), and the brain water content was examined to evaluate brain swelling in the ischemic brain (Figure 3B). Rats pretreated with PNGL (73, 146, 292 mg/kg) had significantly smaller infarcts volumes than those in the MCAO/R group (respectively, 73, * *p* < 0.05; 146, ** *p* < 0.01; 292, ** *p* < 0.01, Figure 3A,C). Moreover, there was a significant increase in brain swellings (brain water content) after 24 h of reperfusion, whereas PNGL (146, 292 mg/kg) pretreatment remarkably reduced the brain water contents (respectively, 146, * *p* < 0.05; 292, ** *p* < 0.01, Figure 3B). Furthermore, NBP (60 mg/kg) and ASP (10 mg/kg) has similar neuroprotective effects against brain swellings and infarcts with a dose-dependent way (Figure 3B,C).

Moreover, the neurological deficit scores were evaluated using the scoring criteria of Zea-Longa. Longa scores suggested that MCAO/R significantly increased neurological deficit scores in rats (## *p* < 0.01, Figure 3D). In comparison with the MCAO/R group, PNGL administration (73, 146, 292 mg/kg) resulted in a significant decrease in neurological deficit scores (respectively, 73, * *p* < 0.05; 146, ** *p* < 0.01; 292, ** *p* < 0.01, Figure 3D). A 146 and 292 mg/kg doses approximately possessed equivalent neuroprotective effects, compared with those in NBP and ASP.

### 3.2. PNGL Alleviates BBB Disruption and Inflammatory Cytokines in MCAO/R Rats

**Figure 4:**
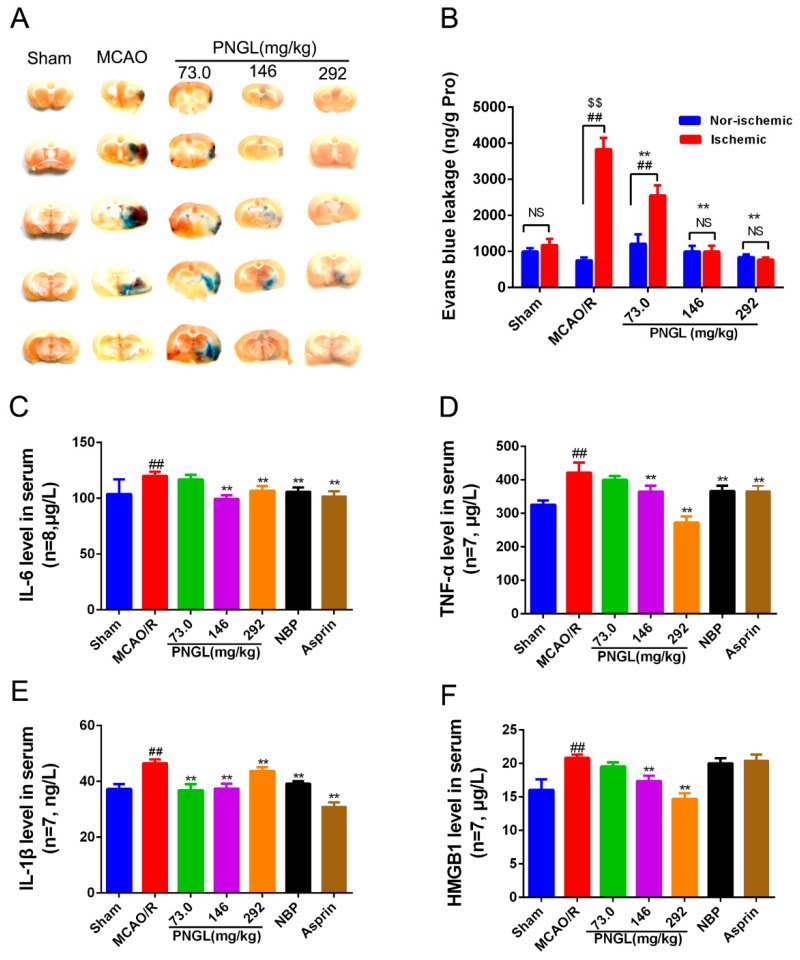
Effects of PNGL on BBB disruption and inflammatory cytokines in MCAO/R rats. PNGL alleviates BBB disruption and inflammatory cytokines in MCAO/R rats. (**A**) Representative images of the Evans blue dye -stained brain sections from the sham-operated or PNGL-treated animals collected 24 h after infarction (4 in each group). (**B**) The Evans blue leakage content of all groups, measured at 632 nm using spectrophotometry (*n* = 5 in each group). (**C**)–(**F**) the IL-6, TNF-α, IL-1 β, and HMGB1 concentrations, determined by ELISA and specific assay kit (*n* = 7–8 in each group). Mean values ± SD; * *p* < 0.05, ** *p* < 0.01 versus MCAO/R group; ## *p* < 0.01, versus sham group; $$*p* < 0.01, Nor-ischemic versus Ischemic; NS means no significance, Nor-ischemic versus Ischemic.

Cerebral I/R injury was reported to exaggerate BBB breakdown in animal stroke models. BBB permeability in MCAO rats was evaluated via the observation of the Evans blue dye (Figure 4A,B). Results showed that Evans blue content in ipsilateral ischemic hemispheres was remarkably increased in the MCAO/R group compared with the sham group (*p* < 0.01, Figure 4A,B). PNGL (73.0, 146, 292 mg/kg) pretreatment significantly decreased the Evans blue leakage content through BBB in ipsilateral ischemic hemispheres (*p* < 0.01, Figure 4B). The non-ischemic hemispheres were not significantly different with the ischemic hemispheres (*p* > 0.05, Figure 4B).

Inflammation has been reported to be involved in the disruption of the BBB. To explore whether PNGL pretreatment could induce anti-inflammatory pattern, ELISA and specific assay kits were used to investigate the expression inflammatory cytokines in serum from MCAO rats after 24 h of reperfusion. The MCAO/R group had significantly higher concentrations of IL-6, TNF-α, IL-1β, and HMGB1 compared to the sham group (*p* < 0.01, Figure 4). In comparison with the MCAO/R group, PNGL (146, 292 mg/kg) pretreatment significantly reduced concentrations of the IL-6, TNF-α, IL-1β, and HMGB1 in the serums (*p* < 0.01, Figure 4C–F). PNGL (73 mg/kg) showed no significant differences on the IL-6, TNF-α, and HMGB1 concentrations with MCAO/R groups (*p* > 0.05, Figure 4C,D,F). Additionally, NBP (60 mg/kg) and ASP (10 mg/kg) has similar decreases in the IL-6, TNF-α, and IL-1 β, concentrations in the serums (*p* > 0.05, Figure 4). Moreover, PNGL (292 mg/kg) pretreatment significantly decreased the ICAM-1 concentration in the serums (*p* < 0.01, Supplementary Material S2A)

### 3.3. PNGL Decreases Neuronal Apoptosis and Loss Caused By Ischemia

**Figure 5:**
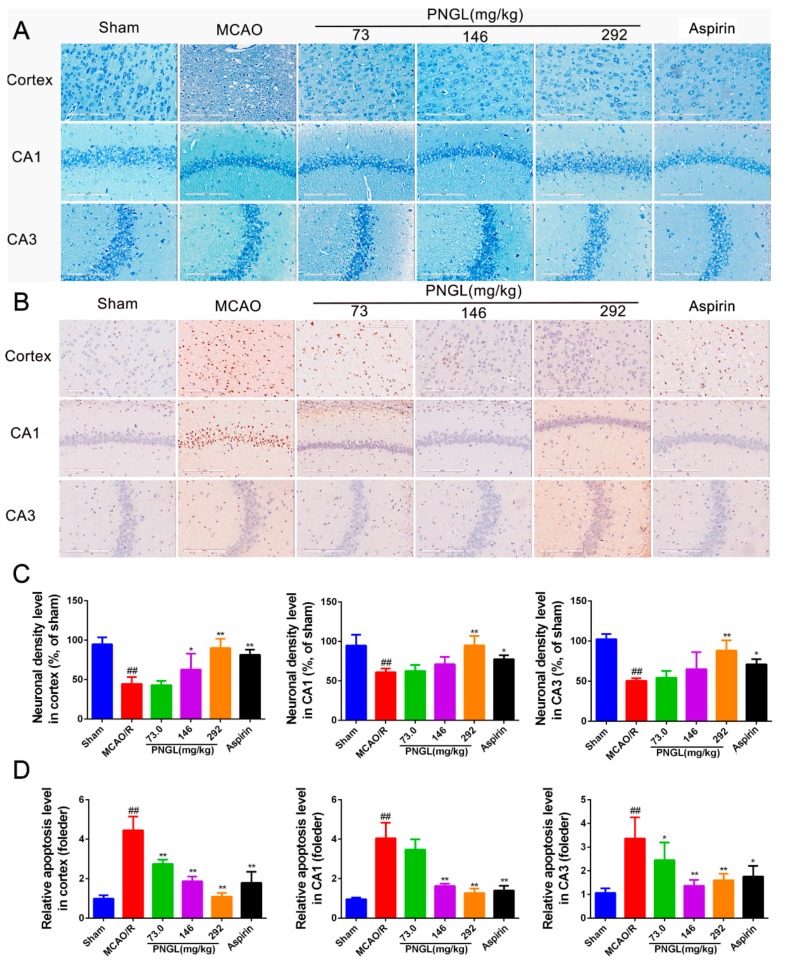
PNGL decreases neuronal apoptosis and neurons loss caused by ischemia. PNGL decreases neuronal apoptosis and neurons loss caused by ischemia in rats. (**A**) Representative images of Giemsa staining performed in hippocampus CA1, CA3 regions, and cortex regions from ischemic brains, measured by an in situ apoptosis detection kit. (**B**) Representative images of TUNEL assay performed in hippocampus CA1 and CA3 regions, and cortex regions. (**C**) The relative neuronal density (%, of sham) in the in hippocampus CA1 and CA3 regions, and cortex regions in all groups (*n* = 4 in each group). (**D**) The relative neuronal apoptotic rate levels in the in hippocampus CA1 and CA3 regions, and cortex regions in all groups (*n* = 3 in each group). Mean values ± SD; * *p* < 0.05, ** *p* < 0.01 versus MCAO/R group; ## *p* < 0.01, versus sham group. Scale bar, 200 μm.

The CA1 and CA3 areas of the hippocampus are commonly considered to be sensitive to ischemic injury [47,48,49,50], and so the CA1 and CA3 areas are used to monitor the neuronal apoptosis and loss. To explore the histopathological changes in the ischaemic brain hemisphere, we performed Giemsa staining. Giemsa staining revealed a change in neuron density. After MCAO/R induction, most neurons exhibited weak staining, which indicated that neurons were diffusely deteriorated, and many neurons were lost in the hippocampus neurons (Figure 5A,C, CA1, *p* < 0.01; CA3, *p* < 0.001; cortex, *p* < 0.01). In contrast, PNGL (146, 292 mg/kg) pretreatment exhibited strong staining and possessed neurons arranged regularly in the hippocampus and cortex (Figure 5A). PNGL (292 mg/kg) presented a significant increase in neuron density (Figure 5C, *p* < 0.01). In addition, PNGL (146 mg/kg) showed similar improvements of nervous density in the hippocampus regions with significant differences (Figure 5C, *p* < 0.01), and ASP (10 mg/kg) were equivalent to those of PNGL in improving neuron density, indicating that PNGL decreases neuronal apoptosis and loss caused by CIRI.

Contrary to Giemsa staining, TUNEL Staining (Figure 5B,D) showed that the relative apoptosis cell levels in the model group was significantly higher than that of the sham group. PNGL (73.0, 146, 292 mg/kg) pretreatment dose dependently markedly decreased neuropathological apoptotic alterations in the brain after 24 h reperfusion following 2 h of MCAO (Figure 5D, * *p* < 0.05, ** *p* < 0.01, *p* < 0.01). Moreover, ASP (10 mg/kg) evidently inhibited the neuronal apoptosis in hippocampus CA1, CA3, and cortex regions, which nearly corresponded to PNGL (146 mg/kg).

### 3.4. PNGL Downregulates Inflammatory Cytokines and Inhibits Microglia Activation in Ischemic Brains

Researches exhibited that the inflammatory factors were induced not only in microglia of the CA1 region in the ischaemic hippocampus but also of the CA3 and DG regions [42,43], so the CA1 region and DG regions were further used to detect the inflammatory response [42,43,47,51,52].

To further explore neuropathological alterations of neurogenic inflammation in ischemic brains from all groups, inflammatory cytokines and microglia activation in ischemic brains were also examined after 24 h reperfusion following 2 h of MCAO. Immunofluorescent results showed that the MCAO/R model rats exhibited more IBA1-positive neurons in the ischemic hippocampus CA1, dentate gyrus (DG), and cortex regions than the sham-operated group (Figure 5A). However, PNGL (146 mg/kg) administration decreased the number of IBA1-positive neurons in the ischemic brain.

**Figure 6:**
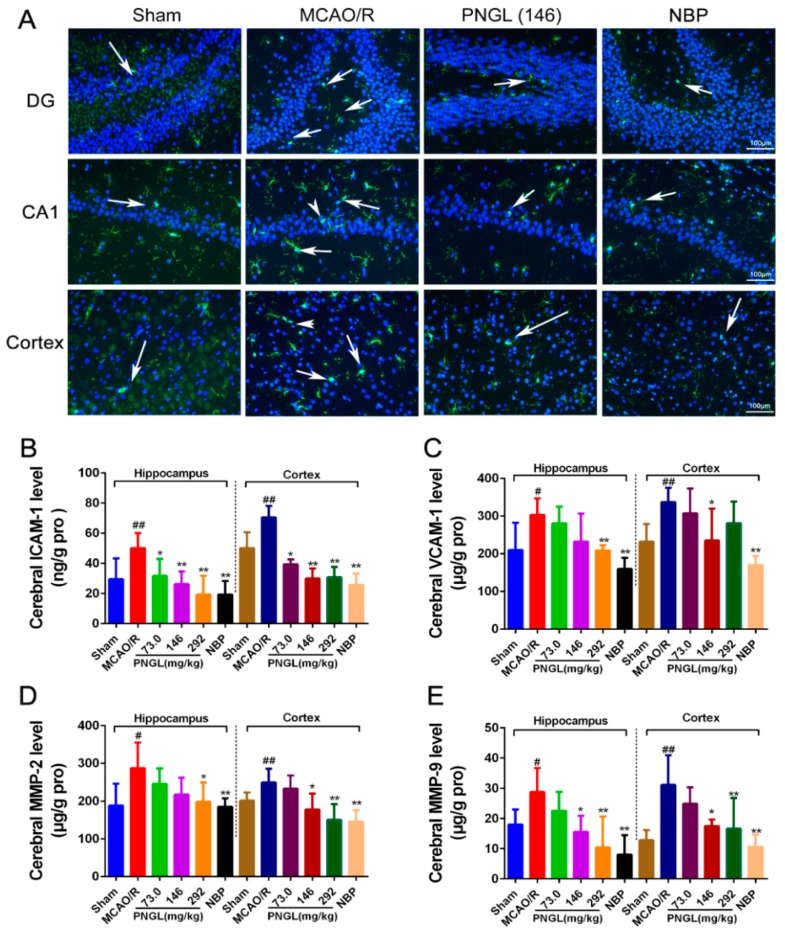
PNGL downregulates inflammatory cytokines and inhibits microglia activation in ischemic brains. PNGL treatment effectively deceased the inflammatory cytokine concentrations, inhibited the microglia activation, and reduced the neuronal loss and apoptosis, thus improving cerebral I/R-induced neuropathological changes by inhibiting neurogenic inflammation. (**A**) Representative images of IBA1-immunopositive microglia (green) with DAPI (blue) staining from hippocampus CA1, dentate gyrus (DG), and cortex regions in rat brains after MCAO/R injury, measured by immunofluorescence. (**B**)–(**E**) The MMP-2, MMP-9, ICAM-1, and VCAM-1 concentrations in hippocampus and cortex in rat brains after MCAO/R injury, determined by ELISA and specific as; (**F**) the IOD of IBA1-immunopositive microglia (*n* = 4–6 in each group). Mean values ± SD; * *p* < 0.05, ** *p* < 0.01 versus MCAO/R group; # *p* < 0.05, ## *p* < 0.01, versus sham group. Scale bar, 100 μm.

Similarly, the MCAO/R group significantly increased the inflammatory cytokines in ischemic brains than did the sham group (*p* < 0.05, Figure 6B–E), including the MMP-2, MMP-9, ICAM-1, and VCAM-1 concentrations in the hippocampus and cortex regions. In comparison with the MCAO/R group, PNGL (73.0, 146, 292 mg/kg) pretreatment dose dependently decreased the inflammatory cytokines in hippocampus and cortex regions. Additionally, PNGL (292 mg/kg) pretreatment significantly reduced the concentrations of MMP-2, MMP-9, and ICAM-1 (*p* < 0.05, Figure 6B,D,E). PNGL (146 mg/kg) pretreatment significantly decreased the VCAM-1, MMP-9, and ICAM-1 concentrations (*p* < 0.05, Figure 6B,C,E). PNGL (73 mg/kg) showed a minor decrease in the inflammatory cytokine concentrations with no significant difference. In additions, NBP (60 mg/kg) has similar decreases of the inflammatory cytokine concentrations in ischemic brains (*p* < 0.01, Figure 6 B–E).

Taken together, these results indicated that PNGL treatment effectively deceased the inflammatory cytokine concentrations, inhibited the microglia activation, and reduced the neuronal loss and apoptosis, thus improving cerebral I/R-induced neuropathological changes by inhibiting neurogenic inflammation.

### 3.5. PNGL Inhibits NF-Κb Signaling Pathway

**Figure 7:**
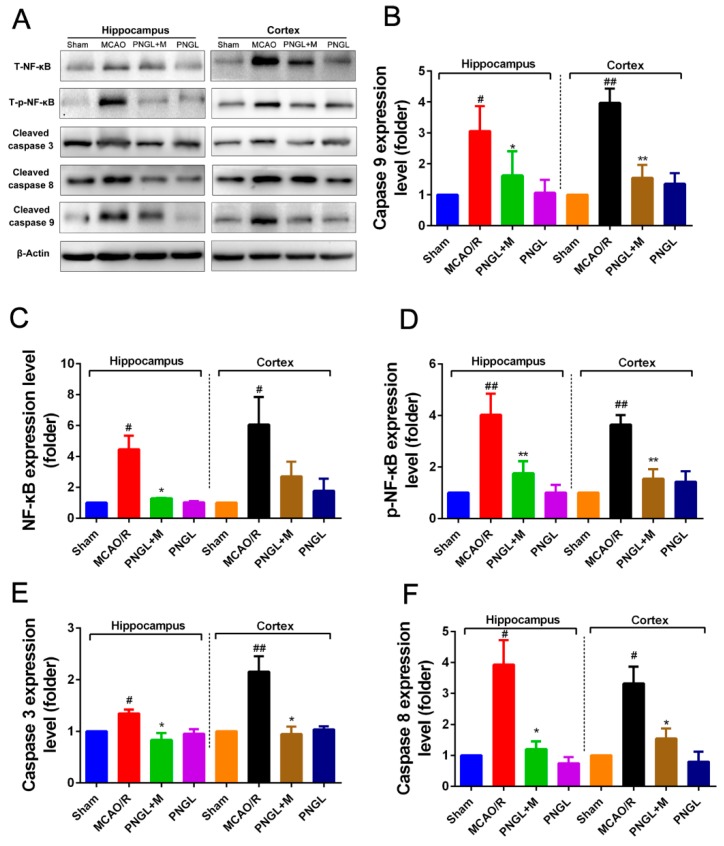
Effects of PNGL on the expression levels of phosphorylated NF-κB. PNGL regulated downstream Caspase 3/8/9 signaling pathway in the ischemic brain. PNGL inhibited the NF-κB activation, it regulated neuronal apoptosis and neuroinflammation reactions caused by CIRI. (**A**) The protein bands of the NF-κB regulated downstream Caspase 3/8/9 signaling pathways in the ischemic brain, which were examined by western blot analysis. (**C**) the relative NF-κB expression level; (**D**) the relative expression levels of the phosphorylated NF-κB; (**E**) (**F**) (**B**) The relative expression levels of its downstream Caspase 3/8/9, respectively, quantified and analyzed by using Gel-Pro analyzer software. Mean values ± SD (*n* = 3); Mean values ± SEM; * *p* < 0.05, ** *p* < 0.01 versus MCAO/R group; # *p* < 0.05, ## *p* < 0.01, versus sham group.

Considering that the activation of NF-κB signaling pathway is related to inflammatory reactions, we finally investigated whether PNGL can inhibit the NF-κB signaling pathway to reduce inflammatory, contributing to the attenuation of cerebral I/R injury. Hence, western blot analysis was used to examine the NF-κB and it regulated downstream Caspase 3/8/9 expression in the hippocampus and cortex regions from ischemic brains. Compared to the control group, the MCAO/R group significantly increased the expressions of the total NF-κB and phosphorylated NF-κB in the hippocampus and cortex regions from ischemic brains (*p* < 0.05, *p* < 0.01, respectively, Figure 7A,C,D). PNGL (146 mg/kg) pretreatment notably inhibited the expressions of the total NF-κB and phosphorylated NF-κB in cerebral ischemia brains (*p* < 0.05, *p* < 0.01, respectively, Figure 7B,C). Similarly, the increase expressions of its downstream caspase 3/8/9 proteins induced by MCAO/R was strikingly abrogated by PNGL pretreatment (hippocampus, *p* < 0.05; cortex, *p* < 0.01, Figure 7B,E,F). These findings suggested that PNGL inhibited the NF-κB activation, it regulated neuronal apoptosis and neuroinflammation reactions caused by CIRI. In additions, no significant change in the expression of cleaved caspase 9 was observed in hippocampus.

### 3.6. PNGL Regulates Mapks Signaling Pathway

**Figure 8:**
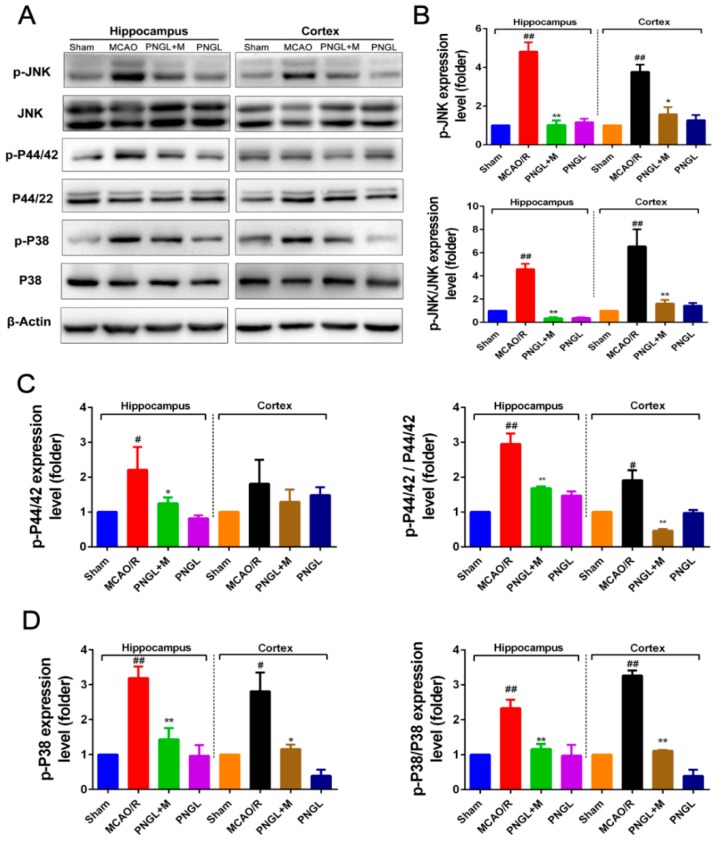
Effects of PNGL on the expression levels of phosphorylated P44/42, JNK1/2, and P38 of MAPKs signaling pathway in the ischemic brain. PNGL downregulated the expression levels of phosphorylated P44/42, JNK1/2, and P38 of MAPKs. (**A**) The protein bands of phospho-ERK, phospho-P38, and phospho-JNK, respectively, in the ischemic brain sections were examined by western blot analysis. (**B**)–(**D**) The relative expression levels of phosphorylated P44/42, JNK1/2, and P38, respectively, were quantified and analyzed by using Gel-Pro analyzer software. Mean values ± SEM (*n* = 3); *p* < 0.05, ** *p* < 0.01, versus MCAO/R group; # *p* < 0.05, ## *p* <0.01 versus sham-operated group.

The MAPKs signaling pathways plays a critical protective role against OGD/R and MCAO/R-induced apoptosis and inflammation. Western blot analysis at 24 h following reperfusion showed, the expression levels of total phosphorylated JNK1/2 (p-JNK1/2), phosphorylated P44/42 (p-P44/42), and phosphorylated P38 (p-P38) significantly increased in the MCAO/R model (*p* < 0.01, *p* < 0.05, *p* < 0.01, respectively, Figure 8). Comparatively, PNGL (146 mg/kg) pretreatment markedly reduced the total phosphorylation levels of the JNK1/2, P44/42 and P38 (*p* < 0.01, *p* < 0.05, *p* < 0.01, respectively, Figure 8 B–D). Additionally, the ratio between the phosphorylated and the non-phosphorylated expression levels of MAPKs showed similar results (*p* < 0.01, *p* < 0.05, *p* < 0.01, respectively, Figure 8 B–D). Moreover, no significant differences were observed in the expression of cortex p-P44/42, total P44/42, total JNK1/2, and total p38 MAPK among all experimental groups (*p* > 0.05, Supplementary Material S2B–D), indicating that PNGL may downregulate the phosphorylation levels of the MAPKs, thus decreasing neuroinflammation after ischemia by modulating innate and adaptive immunity.

### 3.7. PNGL Inhibits HMGB1 Expression and Its HMGB1-Triggered Inflammation

It was proven that HMGB-1 triggers the NF-κB, MAPKs, STAT3, and AP-1 signaling via activating TLR4 signaling, which in turn increases innate and acquired immunity, and further heightens the post-ischemic neuroinflammation response. Accordingly, intercepting the HMGB-1/TLR4 signaling pathway is a promising therapeutic strategy for alleviating cerebral stroke injury and limiting neuroinflammation.

**Figure 9:**
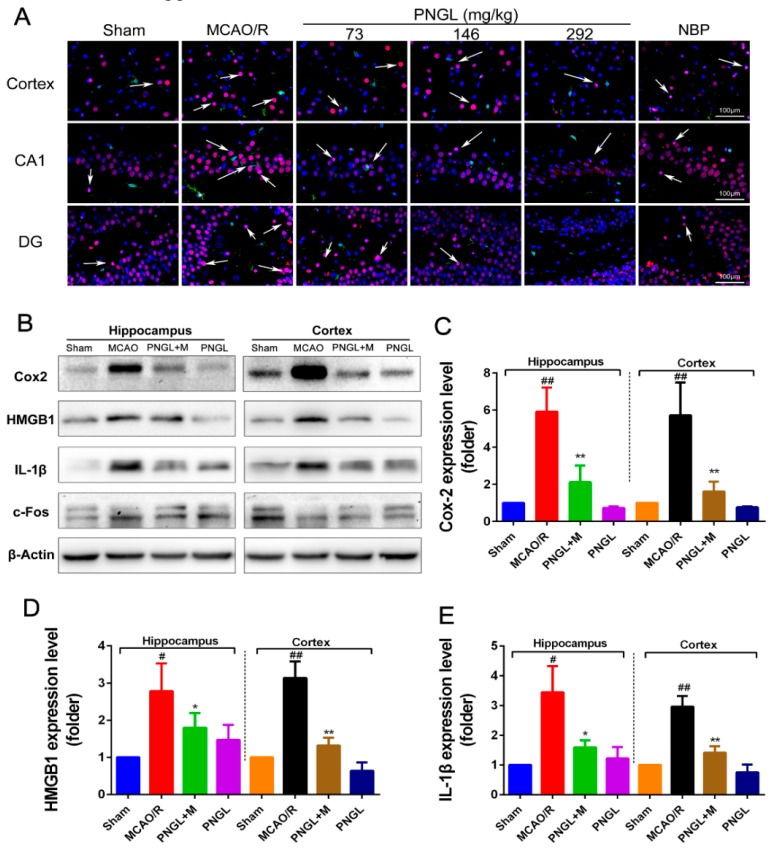
Effects of PNGL on HMGB1 expression and its HMGB1-triggered inflammation in ischemic brains. PNGL treatment effectively deceased the HMGB1 expression and its HMGB1-triggered inflammation, thus improving cerebral I/R-induced neuropathological changes by inhibiting neurogenic inflammation. (**A**) Representative images of HMGB1-immunopositive neurons (red) and IBA1-immunopositive microglia (green) with DAPI (blue) staining in hippocampus CA1, dentate gyrus (DG), and cortex regions in rat brains after cerebral I/R, measured by immunofluorescence; scale bar, 100 μm. (**B**) The protein bands of HMGB1, and the HMGB1-triggered inflammation IL-1β, Cox2 and c-Fos in the ischemic brains were examined by western blot analysis. (**C**)–(**E**) The relative expression levels of HMGB1, IL-1β, and Cox2, respectively, quantified and analyzed by using Gel-Pro analyzer software. Mean values ± SEM; * *p* < 0.05, ** *p* < 0.01 versus MCAO/R group; # *p* < 0.05, ## *p* < 0.01, versus sham group. Scale bar, 100 μm.

As shown in Figure 9, the total HMGB1 level in ischemic brain was notably upregulated in the model group compared with the sham control group (*p* < 0.05, *p* < 0.01, Figure 9A,D), which was obviously downregulated by PNGL (146 mg/kg) pretreatment in hippocampus and cortex regions (*p* < 0.05, *p* < 0.01, Figure 9A,D). Meanwhile, MCAO/R markedly increased the HMGB1-triggered inflammation protein Cox2 and IL-1β expression levels, whereas conversely PNGL (146 mg/kg) pretreatment markedly reversed the increased changes (*p* < 0.05, *p* < 0.01, Figure 9 B,C,E). Hence, our findings suggested that PNGL may decrease HMGB-1 expression in hippocampus and cortex regions and inhibit its HMGB1-triggered inflammation in ischemic brain cells.

## 4. Discussion

Ischemic stroke remains one of the leading causes of death worldwide, which mainly caused by cerebral ischemia and reperfusion injury [1,3,4]. Numerous studies on stroke have been performed in recent decades, but the pathogenesis of ischemic stroke has not been fully elucidated, and few medicines are available [53,54]. Therefore, the development of novel therapeutic options and strategies is urgently needed to limit injury after cerebral infarction. In the current study, we initially proved that PNGL exerted neuroprotective effects against cerebral ischemia and reperfusion injury in middle cerebral artery occlusion/reperfusion (MCAO/R) model rats, and provided a novel mechanism by which PNGL may ameliorate cerebral I/R injury.

PNGL, as the total saponins of Panax notoginseng stem and leaf, has already been shown to exert potent neuroprotective and anti-apoptotic properties. Since the first beneficial effect of PNGL was detected on nervous diseases, several reports have not only revealed its diverse pharmacological properties but have also raised some speculative proposals concerning its mechanism of action. Moreover, currently there had been no clear evidence on whether PNGL has neuroprotective effects against cerebral I/R injury. Meanwhile, our research found that PNGL pretreatment improved the neurological functions, attenuated the brain swelling, and reduced the cortex infarct volume in MCAO/R rats (Figure 3). Additionally, PNGL may evidently alleviate BBB disruption (Figure 4), improve nervous density, and decreases neuronal apoptosis and neuron loss caused by cerebral I/R injury (Figure 5). All of these results proved neuroprotective effects of PNGL against cerebral I/R injury.

Immunity and inflammation are key elements that contribute to the pathobiology of stroke, and its caused-CI/RI and the secondary damage to brain tissues are closely associated with immunity and inflammation responses [5,6,7,8]. Previous research has shown that immunity and inflammation are integral parts of the pathogenesis of ischemic stroke [55,56,57]. Inflammatory signaling is activated and completed by blood-borne leukocytes that penetrate the brain during the ischemic phase [2]. Once ischemia/reperfusion occurs, HMGB1secretion, ROS production, glutamate toxicity, and Ca^2+^ overload promotes the activation of complements, platelets and endothelial cells, activates inflammatory transcription factors and the release inflammatory signals [57], and generates inflammatory cytokines and pro-inflammatory mediators including TNF-α, IL-1β, IL-6, ICAM-1, IFN-γ, IL-17, IL-23, MIP-1α, MIP-2, MMP-2, MMP-9, and anti-inflammatory IL-10 in ischemic brain [55,56,57]. In our research, compared with the sham group, the levels of the inflammatory mediators TNF-α, IL-1β, IL-6, ICAM-1, MMP-2, MMP-9, and HMGB1 significantly increased in the MCAO /R group (Figure 4 and Figure 6). Interestingly, PNGL pretreatment markedly downregulated these concentrations of the inflammatory mediators indicating that the neuroprotective effects of PNGL may be related with inhibition of neurogenic inflammation caused by brain ischemia.

HMGB1 is a nuclear DNA-binding protein and an important DAMP, which activates TLR2/4 and RAGEs signaling following rapid translocation to the cytoplasm or release from dying cells after cerebral ischemia [9,10]. Additionally, cellular HMGB1 binds to pattern recognition receptors of microglia and subsequently leads to synthesis and release of pro-inflammatory mediators, aggravating neuronal injury and BBB disruption [20,58]. Subsequently, the activation of TLR4 signaling at the plasma membrane triggers NF-κB, MAPK, STAT3, and AP-1 signaling, which in turn increases innate and acquired immunity and further heightens the post-ischemic neuroinflammation response [10,59]. Accordingly, intercepting the HMGB1/TLR4 signaling pathway is a promising therapeutic strategy for alleviating cerebral stroke injury and limiting neuroinflammation. Our data indicated MCAO/R induced an obvious increase of the HMGB1 concentrations in serums and expressions in ischemic brains (Figure 4 and Figure 9). Consequently highly released HMGB1 triggered post-ischemic neuroinflammation, and there was also a significant up-regulation of IL-6, TNF-α, IL-1β, ICAM-1, MMP-2 MMP-9 (Figure 6A, Figure 9A,B), and Cox2 accompanied by microglia activation (Figure 6), which was consistent with the previous existing reports [6,7]. However, PNGL (73, 146, 292 mg/kg) pretreatment markedly decreased HMGB1 expression in ischemic brains and concentrations in serums, inhibited the HMGB1-triggered inflammation, and downregulated the inflammatory cytokine levels (Figure 6A, Figure 9A,B).

Further research results suggested PNGL pretreatment dose-dependently inhibited the HMGB-1/TLR4 signaling-triggered downstream proteins including MAPKs, NF-κB, and cleaved caspase 3/8 [6,7,8], suggesting that the immunomodulatory effect of PNGL was associated with the inhibition of HMGB1-triggered inflammation, the NF-κB, and MAPK signaling pathways. Consistent with the current reports [6,7,8], there was a significant upregulation of p-P44/42, p-JNK1/2, and p-p38 MAPK in MCAO/R rats exposed to 2 h of MCAO and 24 h of reperfusion, and it also markedly caused the NF-κB activation and the p-NF-κB level increase. Interestingly, PNGL administration (146mg/kg) significantly downregulated the levels of p-P44/42, p-JNK1/2 and p-p38 MAPK (Figure 8), and also inhibited expressions of the total NF-κB and phosphorylated NF-κB in ischemic brains (Figure 7). No change in the expression of P44/42, JNK1/2, and P38 was observed. These studies indicated that the protective effects of PNGL against neuronal apoptosis could be associated with inhibiting HMGB1-triggered inflammation, suppressing the activation of MAPKs and NF-κB, and thus improved cerebral I/R-induced neuropathological changes. In additions, PNGL decreases neuronal apoptosis and loss caused by cerebral I/R injury.

These findings, together with our results, supported the neuroprotective effects of PNGL against cerebral I/R injury and neuronal apoptosis via decreasing the HMGB levels, inhibiting HMGB1-triggered inflammation, reduced the production of pro-inflammatory mediators, and suppressing the activation of MAPKs and NF-κB signaling pathways. However, neuroprotective effects of PNGL has not been convincingly confirmed by in vitro models of ischemic brain, and the mechanisms was not evidently sufficient to explain on how PNGL may regulate the subsequent immune and neuroinflammatory responses caused by the HMGB1/TLR2/4 pathway. Hence, further investigations are needed to elucidate the mechanisms more deeply and to investigate the clinical applications of PNGL.

Nevertheless, our results suggest that PNGL represents a potential treatment value for cerebral ischemic infarctions, and works through a mechanism of HMGB1-triggered inflammation inhibition through the MAPK and NF-κB pathway

## 5. Conclusions

In summary, this study supported the neuroprotective effects of PNGL on cerebral I/R injury, and the potential mechanisms may be largely associated with the inhibition of HMGB1-triggered inflammation, reduction of pro-inflammatory mediators including IL-6, TNF-α, IL-1 β, MMP-9, MMP-2, and ICAM-1, and attenuation of neuronal apoptosis and loss caused by ischemia via suppressing the activation of MAPKs and NF-κB signaling pathways. Although further studies are needed to elucidate the roles of MAPK signaling pathways in the cross talk between pro-inflammatory mediators and apoptosis, our findings may represent a novel mechanism of PNGL in focal cerebral I/R injury in rats, and provide new insights into therapeutic targets for ischemia stroke patients.

## Supplementary Materials

The following are available online at https://www.mdpi.com/2218-273X/9/10/512/s1, Figure S1: The chromatograms and the chemical fingerprinting of PNGL established by the high performance liquid chromatography (HPLC). Figure S2: Effects of PNGL on the ICAM-1 concentration in serum, the JNK, P44/42, and P38 expression in MCAO/R rats.
