# Supplementary material for: HMGB1-triggered inflammation inhibition of notoginseng leaf triterpenes against cerebral ischemia and reperfusion injury via MAPK and NF-κB signaling pathways
Source: Biomolecules. 2019 Sep 20;9(10):512. doi: 10.3390/biom9100512 (PMC6843331; doi:10.3390/biom9100512)
Supplement: Supplementary file 1 [file biomolecules-09-00512-s001.pdf]

Supplementary materials

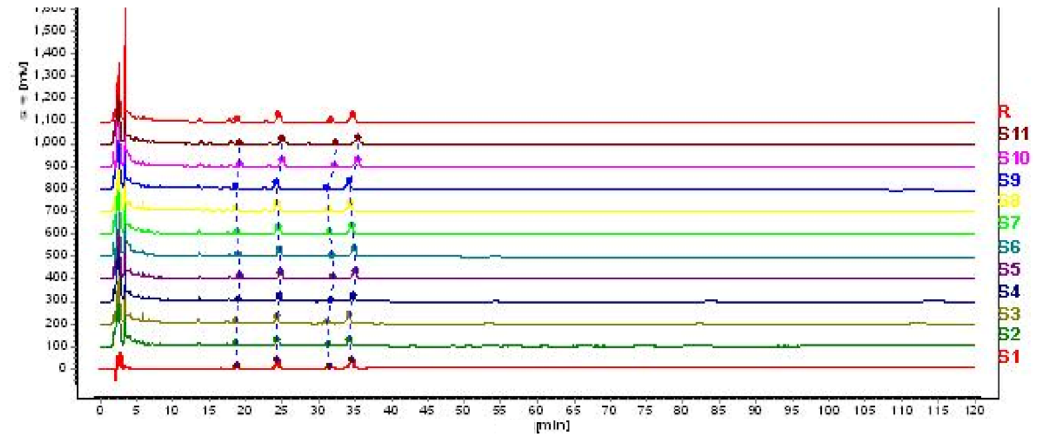

Figure S1. The chromatograms and the chemical fingerprinting of PNGL established by the high performance liquid chromatography (HPLC). The chemical fingerprinting from elven batches of PNGL samples.

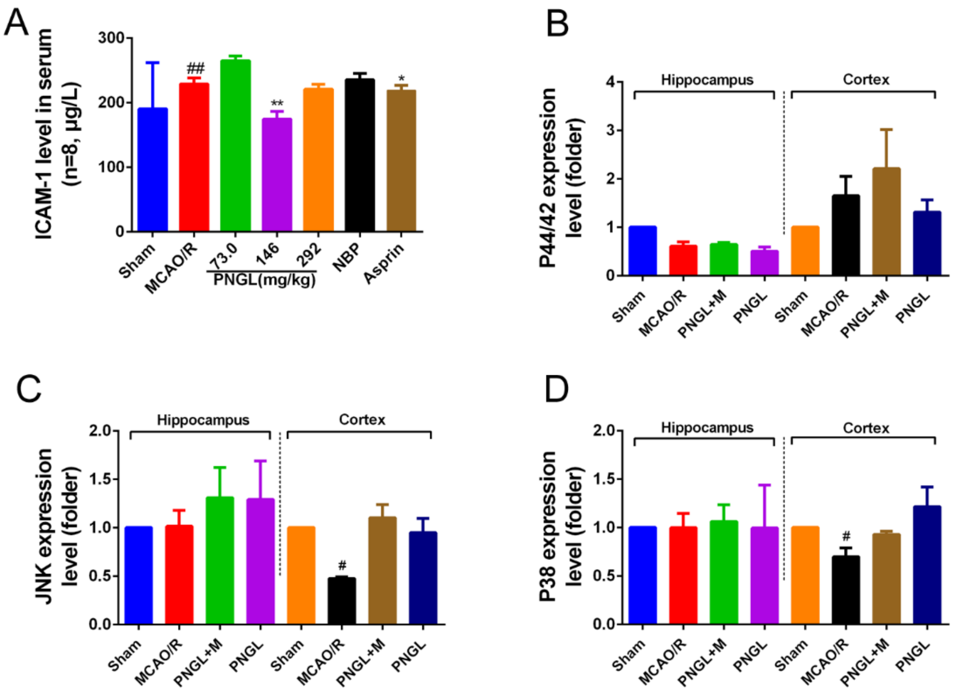

Figure S2. Effects of PNGL on the ICAM-1 concentration in serum, the JNK, P44/42, and P38 expression in MCAO/R rats. PNGL alleviates BBB disruption and inflammatory cytokines in MCAO/R rats. **(A)** The ICAM-1 concentration in serum. **(B) (C) (D)** The JNK, P44/42, and P38 expression (n = 3-4 in each group), measured at 632 nm using spectrophotometry. Mean values ± SEM; * $p < 0.05$, ** $p < 0.01$ versus MCAO/R group; # $p < 0.05$, ## $p < 0.01$, versus sham group.
